# Placental mesenchymal stem cells of fetal and maternal origins demonstrate different therapeutic potentials

**DOI:** 10.1186/scrt436

**Published:** 2014-04-10

**Authors:** Yongzhao Zhu, Yinxue Yang, Yaolin Zhang, Guiliang Hao, Ting Liu, Libin Wang, Tingting Yang, Qiong Wang, Guangyi Zhang, Jun Wei, Yukui Li

**Affiliations:** 1 Institute of Stem Cell Research, General Hospital of Ningxia Medical University, Yinchuan 750004, China; 2 Key Laboratory of the Ministry of Education for Conservation and Utilization of Special Biological Resources in Western China, College of Life science, Ningxia University, Yinchuan 750021, China; 3 Key Laboratory of Fertility Preservation and Maintenance, Ministry of Education, Ningxia Medical University, Yinchuan 750004, China; 4 School of Laboratory Medicine, Ningxia Medical University, Yinchuan 750004, China

## Abstract

**Introduction:**

Therapeutic potentials of mesenchymal stem cells (MSCs) from different sources have been evaluated in pre-clinical and clinical settings. Although MSCs from different sources share MSC-specific characteristics and functions, inconsistent or controversial results of pre-clinical and clinical applications of such cells are frequently reported. This may be partially due to the fact that MSCs isolated from different origins may differentially express some functions not typical for MSCs, and hence have different therapeutic potentials. The aim of this study is to investigate the differences in human placental MSCs (P-MSCs) of fetal and maternal origins in the aspects of clinical importance.

**Methods:**

P-MSCs of fetal and maternal origins isolated from normal term placentas were characterized for their typical phenotype as well as their expression of receptors and growth factors of clinic interests. P-MSCs that preferentially express hepatocyte growth factor (HGF) and CD200 were evaluated for their therapeutic potentials in models of angiogenesis and allogeneic skin transplantation, in comparison with their HGF and CD200 negative partners.

**Results:**

Although all P-MSCs express typical MSC phenotype, fetal but not maternal P-MSCs express high levels of CD200 and HGF. Compared with HGF and CD200 negative P-MSCs, HGF and CD200 positive cells demonstrated significantly high potentials in promoting angiogenesis *in vitro* and increasing immunosuppressive function *in vivo*. These therapeutic potentials were at least in part due to their differences in HGF and CD200 expression, respectively.

**Conclusions:**

We conclude that MSC origins may have significant impact on the therapeutic potentials of such cells, and should be taken into consideration in clinical applications.

## Introduction

Mesenchymal stem cells (MSCs) are considered one of the most promising cell types for therapeutic applications and are, hence, most intensively evaluated in pre-clinical and clinical settings. The characteristics that demonstrate MSC’s high therapeutic potentials include the potential for multiple lineage differentiation [1], the ability of secreting growth factors that can promote cell growth and tissue repair [2]; the function of immunomodulation and immunosuppression [3]; and the limited expression of major histocompatibility complex (MHC) II and co-stimulating molecules which allow MSCs to be used across MHC barriers [4]. Equally noteworthy, not all these characteristics are equally expressed in MSCs of different origins, or in MSCs prepared under different conditions. For instance, CD200, a cell surface molecule mediating an immunosuppressant signal, is expressed in significant levels in some bone marrow-derived MSCs but marginal levels in others, and almost undetectable in MSCs of umbilical cord blood origin [5]. Also, matrix metalloproteinases (MMPs), the molecules that mediate multiple cell-cell interactions and functions of MSCs, are expressed by MSCs in high levels under inflammation conditions but not under normal physical conditions [6]. Such differences may have significant impact on the outcome of clinical treatments when the MSCs are used for cell therapies. For example, hepatocyte growth factor (HGF) is expressed by some MSC cultures [7]. Standal *et al*. [8] reported that HGF decreased bone morphogenetic protein (BMP)-induced osteoblast activity and, hence, increased bone loss, in multiple myeloma (MM) bones, and Xu *et al*. [9] reported that bone marrow-derived MSCs (BM-MSCs) promoted MM cell growth, protected MM cells from drug-induced apoptosis, and shortened the life span of MM-bearing mice, while Li *et al*. [10] demonstrated that placenta-derived MSC-like cells prevented bone loss, stimulated bone formation and suppressed growth of MM in mouse bone. Similar inconsistencies of the therapeutic effects of MSCs from different origins or different preparations on tumor growth have been well documented [11]. These studies together support a notation that MSCs of different origins or prepared under different conditions may express different functions and have different therapeutic potentials.

Of the different sources of MSCs so far investigated, human term placentas have drawn increased interest in recent years [12,13], due mainly to their non-invasive donor procurement and large MSCs supply, in addition to their sharing the basic properties with BM-MSCs [14-17]. A human placenta contains MSCs of both fetal and maternal origin. We [18] and others [13,19,20] have previously isolated and characterized fetal and maternal MSCs from human placentas. Compared with placental MSCs (P-MSCs) of maternal origins, fetal MSCs have demonstrated stronger immunomodulatory function and higher osteogenic differentiation potential [13,20]. However, the defined properties of these cells relevant to therapeutic applications are still poorly understood. In the present report, we tested the differences between P-MSCs of fetal and maternal origins in the aspects of immunosuppressant molecule expression, growth factor secretion, and functions in stimulating angiogenesis and modulating immunosuppression. The results suggest that MSC origins may have significant impact on the therapeutic potentials of such cells, and should be taken into consideration in clinical applications.

## Materials and methods

### Animals

C57BL/6 and Vr:CD1(ICR) mice were obtained from the Surgical Experiment Center of Ningxia General Hospital Affiliated Ningxia Medical University. All animal studies were performed with a protocol approved by the committee of animal care and use at the Ningxia Medical University.

### P-MSCs cell isolation and culture

Human full-term placentas were obtained from healthy mothers at the time of routine elective caesarean section in the Affiliated Hospital of Ningxia Medical University. Informed consent was obtained from each mother prior to delivery. Human placental tissues were collected with a protocol approved by the Ethics Committee for the Conduct of Human Research at Ningxia Medical University. The Human Research Ethics Committee at Ningxia Medical University approved this study. Fetal and maternal P-MSCs were isolated and genetic origin verified as described previously [18]. The cells were cultured in Dulbecco’s Modified Eagle Medium (DMEM) supplemented with 10% fetal bovine serum (FBS), 2 mM L-glutamine and 50 μg/ml gentamycin (Invitrogen, Carlsbad, CA, USA). This medium is referred to as P-MSC medium. All cultures were maintained at 37°C in a humidified incubator with 5% CO_2_. At about 90% confluence, the cells were passaged after detachment with TrypLE™ Express (Invitrogen). All the studies were performed with five passages of the established P-MSCs cultures.

### Flow cytometry

P-MSCs were harvested by TrypLE™ Express treatment and analyzed by flow cytometry with a FACS Calibur flow cytometer (BD Biosciences, San Diego, CA, USA). All monoclonal antibodies used for flow cytometry were obtained from BD Pharmingen (Franklin Lakes, NJ, USA) and prepared in PBS. Cells (1 × 10^6^) in 100 μl were incubated with 10 μl of one of the following antibodies for 20 minutes at room temperature: IgG1-PE, CD34-PE, CD73-PE, IgG2a-FITC, CD14-FITC, CD45-FITC, CD90-FITC, CD105-FITC, CD200-PE or HLA-DR-FITC. After washing, cells were resuspended in 500 μL PBS and phenotyped by flow cytometry analysis.

### RNA isolation and qRT-PCR

Cultured fetal and maternal P-MSCs in passage 5 were washed with PBS and total RNA was isolated with Trizol Reagent (Invitrogen). cDNA was elaborated from RNA using Revert Aid First Strand cDNA Synthesis Kit (Thermo Fisher Scientific, San Diego, CA, USA). Quantitative PCR (qPCR) was carried out using the DyNAmo* Capillary SYBR* Green 2-Step qRT-PCR Kit (Thermo Fisher Scientific). Thermal cycling conditions were 95°C for 10 minutes followed by 40 cycles of 15 s at 95°C, followed by 1 minute at 60°C. We obtained melt data by 30 minutes at 60°C. PCR primer sets for CD200, HGF and GAPDH were: CD200: forward 5′- AATACCTTTGGTTTTGGGAAGATCT-3′, reverse 5′-GGTGGTCTTCAGAGAATTTGTAGTGA-3′; HGF: forward 5′-ATTGCCCTATTTCTCGTTGTG-3′, reverse 5′-GCATTTCTCATCTCCTCTTCC-3′; GAPDH; forward 5′-AACATCATCCCTGCTTCCAC-3′, reverse 5′-GACCACCTGGTCCTCAGTGT-3′.

### ELISA

Equal numbers of fetal and maternal P-MSCs in passage 5 were seeded into 35 mm-Petri dishes, respectively, and cultured in normal medium (as described above) with or without 10 ng/ml IFN-γ (R&D Systems, Inc., Wiesbaden, Germany) or 1 μM poly (I:C) (Invivogen, San Diego, USA) (final concentration) for 72 h. At the end of cell culture, cell numbers were re-counted and supernatants of each culture were collected for ELISA analysis. All ELISA kits were obtained from BOSTER (Wuhan, China). ELISA analysis was performed according to the manufacturer’s protocol. The concentration of each cytokine secreted by P-MSCs is normalized with the cell number at the end of experiments and expressed as pg/ml/10^5^ cells.

### Preparation of P-MSC-conditioned medium

Maternal and fetal P-MSCs in passage 5 were seeded at 1 × 10^6^ cells/100 mm plate with 10 ml P-MSCs medium and cultured for 48 hours. After this cultivation period, medium from each culture was collected and clarified by centrifugation at 1,000 rpm for 10 minutes at 4°C. These media were referred to as maternal and fetal P-MSC-conditioned medium, respectively.

### In vitro angiogenesis assay

Growth factor-reduced Matrigel (BD Biosciences) was thawed at 4°C overnight and spread evenly over 48-well plates pre-chilled at 4°C. The plates were incubated for 30 minutes at 37ºC to allow gel formation. Human umbilical vein endothelial cells (HUVECs) (BIOLEAF, Shanghai, China) were cultured in DMEM with 10% FBS, 50 U/ml penicillin and 50 ug/mL streptomycin (Invitrogen). Cells that reached 80% confluency were collected by trypsinization and centrifugation. Single cell suspensions were prepared in P-MSCs medium, P-MSCs medium plus 20 ng/ml recombinant human growth factor (rhHGF) (R&D System), maternal P-MSC-conditioned medium, fetal P-MSC-conditioned medium, and fetal P-MSC-conditioned medium plus 100 ng/ml anti-human HGF antibody (R&D System). A total of 3.5 × 10^4^ cells in 500 μl of each medium were evenly seeded over a Matrigel-coated well of 48-well plates. After incubation for 12 hours, the cultures were imaged under a microscope and the numbers of capillary tube structures were counted from nine randomly chosen fields for each culture condition.

### Skin transplantation

Vr:CD1(ICR) mice at postnatal Day 3 were used as skin donors, and C57BL/6 mice at age 8 to 10 weeks of age were used as recipients. The donor mice were sacrificed with CO_2_, and then skin grafts of 1.2 cm in diameter were prepared using a surgical puncher. The recipient mice were anesthetized with chloral hydrate (Sangon Biotech, Shanghai, China), and a round incision of 1.2 cm in diameter was made on the dorsal side of each recipient mouse. After the removal of full-thickness skin from the incision sites of the recipient mice, donor skin grafts were transplanted into the sites, and dressed with Vaseline dressings and Band-Aids. At the time of the skin transplantation, the recipient mice were injected (tail i.v.), respectively, with 200 μl PBS, 1 × 10^5^ maternal P-MSCs in 200 μl PBS, 1 × 10^5^ fetal P-MSCs in 200 μl PBS and 1 × 10^5^ CD200 blocked fetal P-MSCs. The CD200 of fetal P-MSCs was blocked by using mouse anti-human PE-conjugated CD200 antibody according to the method derived from Mika Pietila *et al*. [5]. Briefly, fetal P-MSCs were incubated with anti-human CD200 antibody (BD Biosciences) at 20 μl antibody/1 × 10^6^ cells in 100 μl PBS for 30 minutes and then washed twice with PBS and suspended in PBS. Grafts were inspected daily from the third day of transplantation to establish time of rejection.

### Statistical analysis

All statistical analyses were performed using SPSS statistical analysis software (SPSS Inc., Chicago, IL, USA). Results were expressed as mean ± SD for illustration. The statistical significance in differences among treatments was determined using one-way ANOVA with a *post-hoc* least square difference (LSD) test. A *P-*value of less than 0.05 was considered significant.

## Results

### Fetal, but not maternal, P-MSCs preferentially express CD200

**Figure 1:**
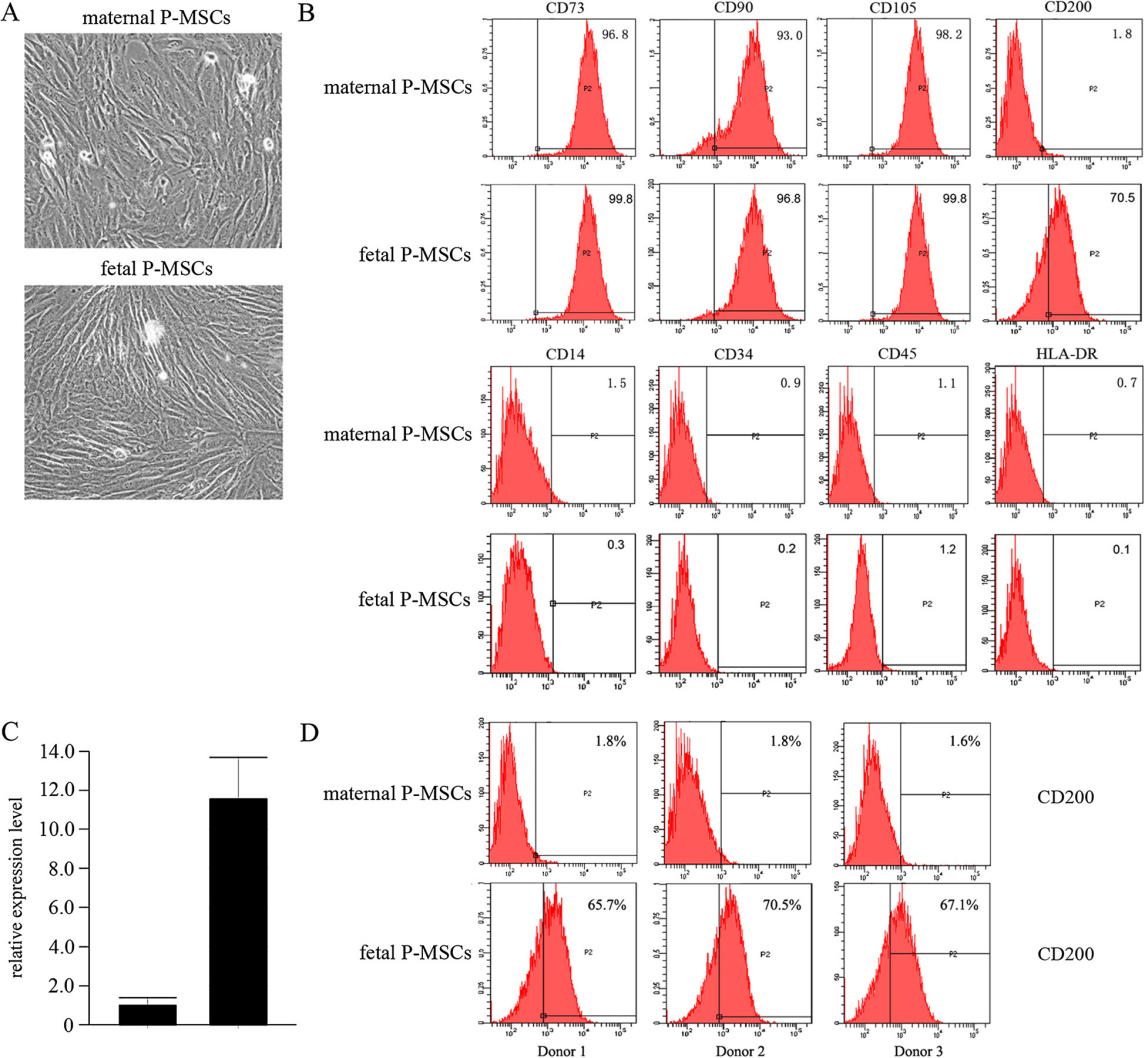
**Fetal and maternal P-MSCs commonly express a MSC-specific phenotype, but differentially express CD200. A**: The morphology of maternal and fetal P-MSCs. **B**: Flow cytometry analysis for PMS-specific phenotype expression by fetal and maternal P-MSCs. Both populations expressed typical MSC phenotype. **C** and **D**: Comparison in CD200 expression in transcript **(C)** and protein **(D)** levels between fetal and maternal P-MSCs. Shown are representatives of three random donors. **: *P* <0.01. MSC, mesenchymal stem cells; P-MSCs, placental mesenchymal stem cells.

The P-MSCs of fetal and maternal origins were isolated and characterized as previously described [18]. For matched comparison, the cells were maintained under the same conditions and exposed to the same treatments throughout the experiments, and the results were compared in pairs of individual placenta donors. Both fetal and maternal P-MSCs express typical MSC morphology and phenotype, they all share the similar spindle-like shape (Figure 1A), as well as being positive for CD73, CD90 and CD105, and negative for CD14, CD45, CD34 and MHC class II receptor HLA-DR (Figure 1B). Since CD200 has been shown to mediate the immunosuppressive function to multiple myeloid cell types, especially to dendritic cells and macrophages [21,22], we tested the expression of this molecule in P-MSCs. As shown in Figure 1B, CD200 positive cells count for about 70% of the fetal P-MSCs population, while being barely detectable in maternal P-MSCs. To confirm this finding, we further isolated P-MSCs from three random donors, and analyzed CD200 expression by qPCR and flow cytometry. As shown in Figure 1C, CD200 transcripts in fetal P-MSCs from the three donors averaged more than 10 times that in maternal P-MSCs (Figure 1C). This finding was further confirmed in protein levels. Flow cytometry demonstrated that CD200^+^ cells were constantly identified in fetal P-MSCs but not in maternal P-MSCs from all three random donors. As CD200 mediates multiple lines of immunosuppressive signals, this result may suggest higher potential of fetal P-MSCs for immunosuppressant treatments.

### Fetal P-MSCs express significantly higher levels of HGF than do maternal P-MSCs

**Figure 2:**
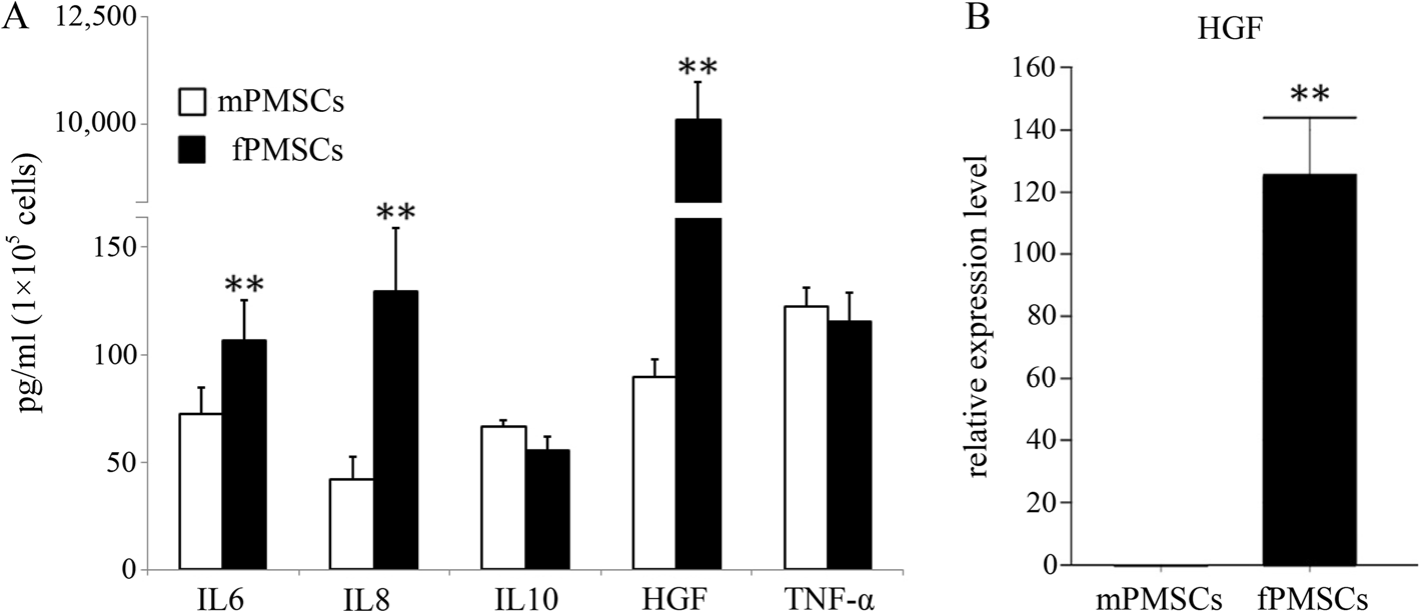
**Differential cytokine expression by fetal and maternal P-MSCs. A**: P-MSC cells from fetal and maternal P-MSCs were seeded in equal numbers and re-counted at the end of culture. Cytokine levels in cell culture supernatants were quantified by ELISA and normalized by cell numbers at the end of the study. **B**: qPCR quantification for HGF expression by fetal and maternal P-MSCs. n = 3. **: *P* <0.01. HGF, hepatocyte growth factor; P-MSCs, placental mesenchymal stem cells.

HGF is an important growth factor that not only promotes cell growth, morphogenesis and tissue regeneration [23], but also exerts a regulatory role in inducing tolerogenic dendritic cells and regulatory T cells [24]. We examined HGF along with interleukins 6 (IL-6), 8 (IL-8), 10 (IL-10) and TNF-α expressed in P-MSCs of fetal and maternal origins in cell number-controlled settings (see Materials and methods). Surprisingly, while the cytokines were expressed in comparable levels in both cell populations, fetal P-MSCs expressed a much higher level of HGF, about 100 times as much as expressed by maternal P-MSCs do (Figure 2A). We further verified this finding by qPCR for transcript expression of HGF. As shown in Figure 2B, a significant difference in HGF transcript expression comparable with protein secretion was observed. This result suggests that a superior benefit may be expected for therapeutic applications of fetal P-MSCs over P-MSCs of maternal origin.

### Fetal and maternal P-MSCs responded to immunostimulation with discrete cytokine pathways

**Figure 3:**
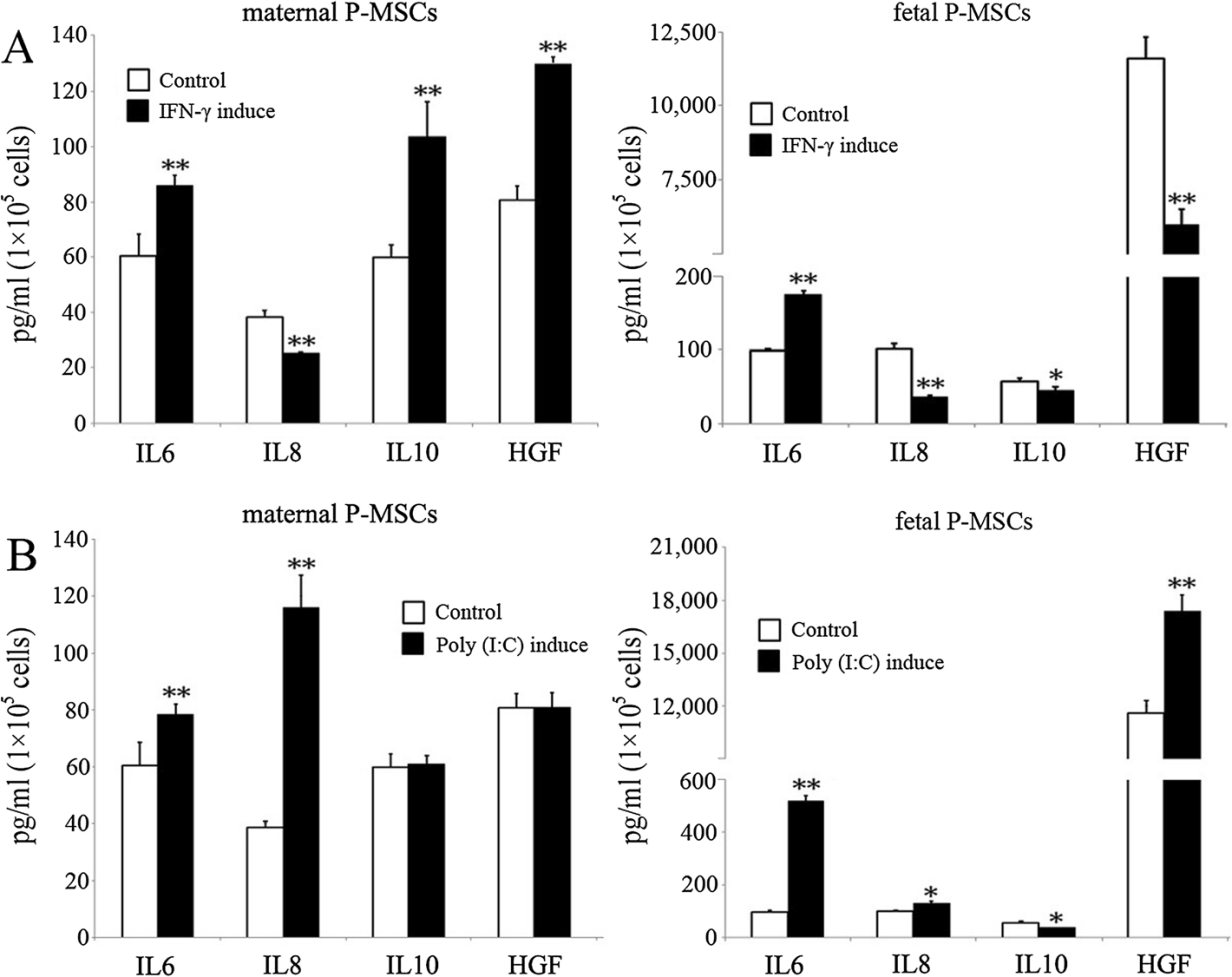
**Cytokine expression by fetal and maternal P-MSCs in response to IFN-γ and Poly (I:C) stimulations.** Equal numbers of cells from fetal and maternal P-MSCs were seeded and stimulated with IFN-γ **(A)** and Poly (I:C) **(B)**. Cytokine levels in cell culture supernatants were quantified by ELISA and normalized by cell numbers at the end of the study. n = 3. *: *P* <0.05, **: *P* <0.01. IFN-γ, interferon gamma; P-MSCs, placental mesenchymal stem cells.

It has been previously reported that interferon gamma (INF-γ) and Toll like receptor-3 ligand poly (I:C) can induce activation and enhance therapeutic potential of MSCs [3,25]. We sought next to elucidate how the P-MSCs of fetal and maternal origin respond to these immune stimuli. Although both cell types responded to the stimuli significantly in the aspect of cytokine induction (Figure 3), they showed discrete cytokine response patterns. Upon INF-γ stimulation, fetal P-MSCs significantly increased IL-6 secretion, and decreased to a lesser but statistically significant extent the secretion of IL-8, IL-10 and HGF; in comparison, in maternal P-MSCs, INF-γ induced significant increases in IL-6, IL-10 and HGF (Figure 3A,B). Considering the immunomodulatory and anti-inflammation properties of IL-10 and HGF, this observation may suggest that INF-γ-priming may be more preferable for maternal P-MSCs than fetal P-MSCs when immunomodulation applications are considered for these cells.

Markedly different from INF-γ, Poly (I:C) stimulation resulted in a significantly increased secretion of IL-8 in maternal P-MSCs but not fetal P-MSCs, and a significantly increased secretion of HGF in fetal but not maternal P-MSCs. (Figure 3C,D). Whether the different responses are due to differentiated expression of TLR-3 in these two cell populations is unknown and need to be further explored.

### Fetal P-MSCs stimulated angiogenesis of HUVECs in vitro

**Figure 4:**
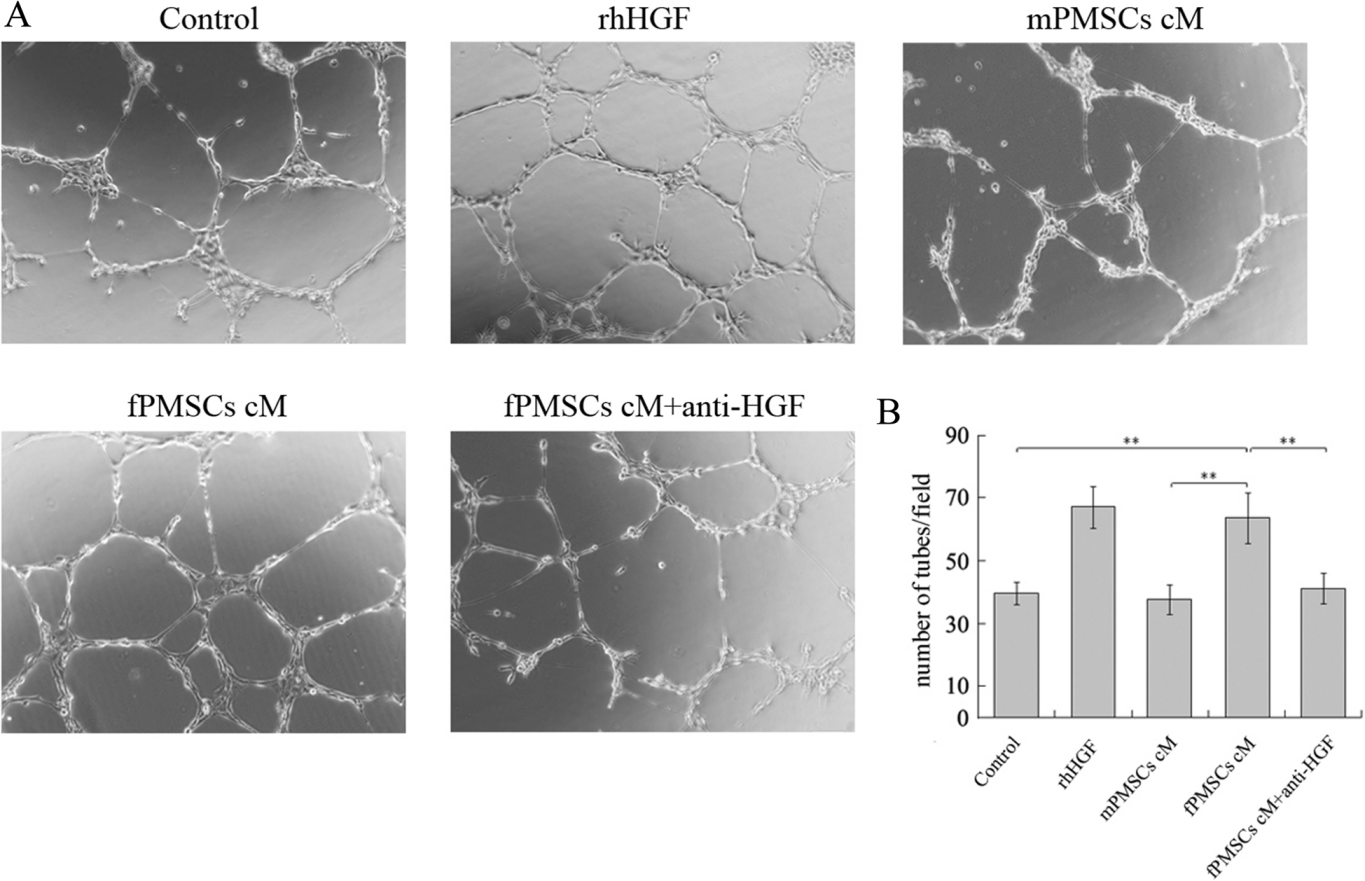
**Fetal P-MSCs (fPMSCs) stimulated angiogenesis *in vitro *in HGF-dependent manner. (A)** Tube formation by HUVECs cultured in P-MSCs medium, P-MSCs medium supplemented with 20 ng/mL rhHGF, maternal P-MSCs conditioned medium (mPMSCs cM), fetal P-MSCs conditioned medium (fPMSCs cM) and fPMSCs cM plus100 ng/mL anti-HGF antibody, respectively. Images are representatives of nine randomly chosen images for each culture condition (100x magnification). **(B)** The average number of tube structures formed in each group as counted from nine randomly chosen images under 40x magnification. **: *P* <0.01. HGF, hepatocyte growth factor; HUVECs, human umbilical vein endothelial cells; P-MSCs, placental mesenchymal stem cells; rhHGF, recombinant human growth factor.

HGF is well known for its function in stimulating angiogenesis of endothelial cells. Having observed that the P-MSCs of fetal origin expressed a much higher level of HGF in both mRNA and protein than P-MSCs of maternal origin (Figure 2), we next tested if this difference would translate into differences in their ability to stimulate angiogenesis. As shown in Figure 4, the human umbilical vein-derived endothelial cells (HUVECs) grew into a limited number of tube structures when cultured in the absence of HGF. When human recombinant HGF was added to the culture, significantly more tube structures were observed. In comparison, when HUVECs were cultured in fetal P-MSCs-conditioned medium, tube formation was enhanced to the level similar to HGF-treated cultures. This enhancement in angiogenesis was not observed when HUVECs were cultured in maternal P-MSCs-conditioned medium, and was inhibited by the addition of anti-HGF antibodies (Figure 4A,B). These results demonstrated that, compared with maternal P-MSCs, fetal P-MSCs expressed an increased ability to stimulate *in vitro* angiogenesis, and it is suggestive, though not conclusive, that this function is dependent on HGF secretion.

### Fetal P-MSCs prolonged skin allograft survival through CD200 expression

**Figure 5:**
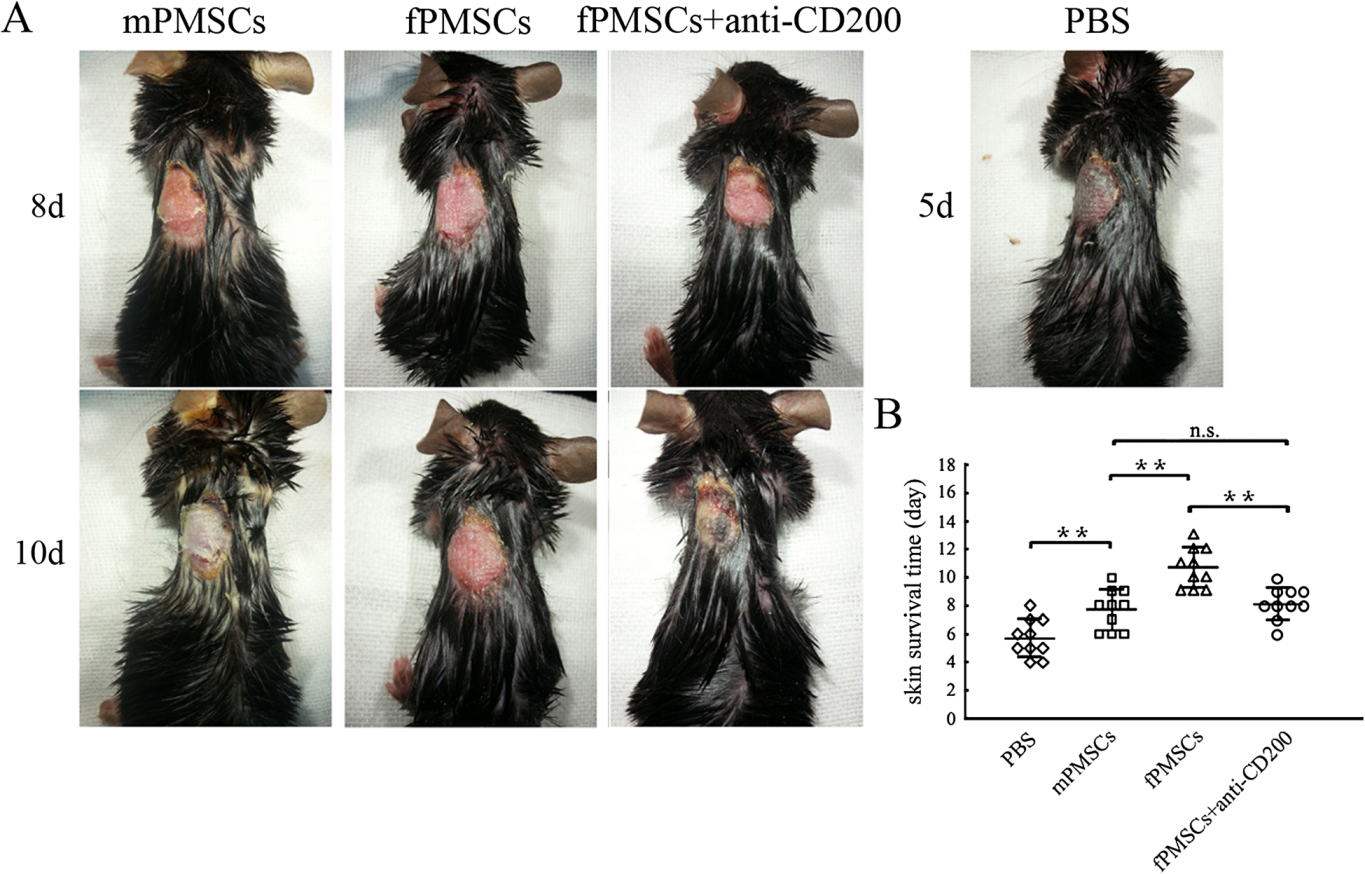
**Fetal P-MSCs (fPMSCs) prolonged skin allograft survival. (A)** Representative images of skin allograft survival in different time points post-transplantation. Different groups show mice treated with PBS, maternal P-MSCs (mPMSCs), fPMSCs and fPMSCs plus anti-CD200 antibodies, respectively. Images are representative of 10 mice for each group. **(B)** Skin allograft survival and average survival times among different mouse groups. n = 10. **: *P* <0.01. PMSCs, placental mesenchymal stem cells.

MSCs are known to have immunosuppressive functions. CD200 is a negative regulator of a number of immune cells, predominantly cells of myeloid origin. Since P-MSCs of fetal origin expressed much higher CD200 than P-MSCs of maternal origin, we reasoned the CD200-expressing cells should have higher immunosuppressive functions. This was tested in a mouse skin allograft model. As shown in Figure 5, without immunosuppressant, skin allografts were rejected within six days. When the recipient mice received an IV injection of fetal P-MSCs, the allografts survived to 10 days on average. When anti-CD200 antibodies were injected into the CD200-expressing cell-treated mice, the graft survival time was reduced to an average of eight days, similar to the survival time in mice treated with CD200-negative cells. These results indicated that fetal P-MSCs prolonged allograft survival, partially but not totally, due to CD200-mediated immunosuppression. Maternal P-MSCs (CD200 negative MSCs) could also increase allograft survival, but to a significantly lower extent than did fetal P-MSCs (CD200 positive MSCs).

## Discussion

MSCs are one of the most promising types of stem cells for recent clinical applications. One existing problem frequently reported with this cell type is the inconsistent or controversial results from pre-clinical and clinical applications, possible reasons, among others, being the different impacts from the sources of the cells, culture systems used and the ratio of different sub-groups with different properties/functions of cells in a given heterogeneous population. In the present study, we started to address this problem by studying paired MSC populations of fetal and maternal origin from the same tissues (individual placentas) and under tightly controlled conditions for their difference in the aspects of clinical relevance. These settings allowed us to define the impact of developmental sources of MSC populations to the cell properties/functions. In this report we have demonstrated that fetal but not maternal P-MSCs express high levels of CD200 and HGF, and respond differently to immunostimulations in terms of cytokine secretion. Since CD200 and HGF have significant clinical relevance, we further demonstrated that, compared with maternal P-MSCs, fetal P-MSCs predominantly express CD200 and HGF prolonged skin allograft survival *in vivo* and stimulated angiogenesis *in vitro*, respectively.

MSCs from multiple different tissues have been isolated and characterized. Although bone marrow MSCs remain the most frequently investigated MSC populations, MSCs from different neonatal tissues have recently drawn special attention [26], due in part to their being ethically non-controversial, easily accessible and MSC-enriched. These MSC origins include amniotic membrane and chorionic membrane [27,28], whole placenta [29], Wharton’s jelly [30] and whole umbilical cord [31]. Comparative studies have indicated that different cell sources may have significant impact on cell properties. For example, Kern *et al*. [32] characterized the MSCs from bone marrow, umbilical cord blood and adipose tissue for their stem cell characteristics under identical *in vitro* conditions, and demonstrated that, although phenotypically similar, these MSC populations exhibited cell source-related heterogeneity in colony frequency, proliferative capability and differentiation potential. Hwang *et al*. [33] demonstrated that MSCs from placenta, cord blood and bone marrow expressed different cytokine profiles. Gaebel *et al*. [34] compared MSCs from umbilical cord blood, adipose tissue and bone marrow for their ability to promote cardiac regeneration, and found that MSCs from different sources showed a different healing performance. In our current study, we demonstrated that P-MSCs from the same placenta but different developmental origins have different properties of therapeutic importance, and we further linked these properties to the differential expression of CD200 and HGF by these two MSC populations. These studies altogether support a note that the sources where the MSCs originate may have a significant impact on the therapeutic potential of the cells.

Among the differences between fetal and maternal P-MSCs identified in our current study, CD200 and HGF are of specific interest. CD200 is a cell surface glycoprotein mediating an immunosuppressant signal, and has been shown to modulate immune responses by multiple myeloid cell types, especially to dendritic cells and macrophages [21,22]. It has been recently reported that cells expressing the CD200 transgene could prolong allograft skin survival [35], and a similar function was demonstrated by using anti-CD200 antibodies [36]. In our present study, we demonstrated that fetal but not maternal P-MSCs expressed CD200 to a significant level, and this expression mediated an anti-rejection function in a mouse skin allograft model. MSCs have been well known for their immunomodulative functions and hence have been tested in multiple clinical settings. Expression of CD200 by MSCs can be thus expected to add clinical benefit when MSCs are applied for immunosuppressive treatments. Pietilä *et al*. has reported that CD200 was detected in some but not all bone marrow-derived MSC populations and was undetectable in umbilical cord blood MSCs [5]. To our knowledge, the present study is the first to report the expression of CD200 in placental MSCs and CD200-mediated immunosuppression by fetal P-MSCs. This finding may provide new insight into the therapeutic potentials of MSCs of neonatal origin. Another finding of particular interest is the preferential expression of HGF by fetal P-MSCs. HGF is a clinically important growth factor that can promote angiogenesis and tissue repair. MSCs have been clinically tested for promoting tissue regeneration and wound healing. Sasaki *et al*. reported that MSCs contributed to wound repair by transdifferentiation into multiple skin cell types [37]. Wu *et al*. demonstrated that MSCs enhance wound healing through differentiation and angiogenesis [38]. Chronic wounds and diabetic ulcers, for example, are generally characterized by hyper-inflammation and impaired angiogenesis. While it is possible that MSCs can exert both roles against both dysfunctions, expression of HGF by these cells can be expected to generate enhanced angiogenesis and wound healing. In our study, HGF is highly expressed by fetal P-MSCs but not their partners of maternal origin, indicating that choosing the appropriate MSC type can be expected to generate better clinical outcomes. In this report, we showed evidence that fetal MSC-conditioned medium, similar to recombinant HGF, stimulated angiogenesis *in vitro* and that the anti-HGF antibody abolished this effect, suggesting the involvement of HGF in MSC-mediated angiogenesis. This experiment, however, could not exclude an off-target effect by the antibody and, hence, did not exclusively prove, though it suggested, that the observed angiogenesis is mediated by HGF expression of MSCs. Compared with the maternal P-MSCs that did not express HGF and failed to stimulate angiogenesis, however, the study clearly demonstrated that fetal but not maternal P-MSCS have the ability to enhance angiogenesis. The ultimate demonstration of the mechanisms underlying MSC-mediated angiogenesis is yet to be explored.

## Conclusions

Compared with maternal P-MSCs, fetal P-MSCs express constitutively higher levels of CD200 and HGF, and this difference can mediate different potentials of immunosuppression *in vivo* and stimulating angiogenesis *in vitro*, respectively. Taking into consideration that, in the present study, all cells and treatments were controlled by the same experimental conditions, and all results were compared between cell population pairs from the same individual donors, the results may suggest that, in general, the sources where the P-MSCs originate may have significant impact on the therapeutic potential of the cells, and, specifically, fetal P-MSCs may be more favorable for applications in cell regeneration, tissue repair and autoimmune disorders where HGF and CD200 may exert a positive effect, and less favorable for applications in immune suppressive cancers where CD200 may mediate breakdown of immunosurveillance and establishment of immune tolerance, or for applications where HGF may enhance tumor-supportive angiogenesis.

## Abbreviations

BM-MSCs: Bone marrow-derived MSCs; DMEM: Dulbecco’s modified Eagles medium; FBS: Fetal bovine serum; FITC: Fluorescein isothiocyanate; fPMSCs: P-MSCs of fetal origin; HGF: Hepatocyte growth factor; HUVECs: Human umbilical vein endothelial cells; IL: Interleukin; INF-γ: Interferon gamma; MHC: Major histocompatibility complex; MM: Multiple myeloma; mPMSCs: P-MSCs of maternal origin; MSCs: Mesenchymal stem cells; PE: Phycoerythrin; P-MSCs: Placental MSCs; qRT-PCR: Quantitative reverse transcription-polymerase chain reaction; rhHGF: Recombinant human growth factor.

## Competing interests

The authors declare that they have no competing interests.

## Authors' contributions

JW, YL and YY conceived and designed the experiments, and drafted the manuscript. YZZ, YLZ, GH, LW, GZ, TL, TY and QW collected samples, performed experiments, acquired data and drafted the manuscript. YZZ analyzed the data and revised the manuscript. YL interpreted data and critically revised the manuscript. All authors read and approved the final version of the manuscript.
